# Six-year survival of reimplanted talus after isolated total talar extrusion: a case report

**DOI:** 10.1186/s13256-017-1517-7

**Published:** 2017-12-15

**Authors:** Jae-Man Kwak, Sung-Keun Heo, Gu-Hee Jung

**Affiliations:** 1 Department of Orthopaedic Surgery, College of Medicine, Gyeongsang National University, and Gyeongsang National University Changwon Hospital, 555 Samjungja-Dong, Changwon-si, 642-160 Republic of Korea; 2 Department of orthopaedic surgery, Gi-Jang Hospital, Busan, Republic of Korea; 3 Department of Orthopaedic Surgery, Asan Medical Center, University of Ulsan, Seoul, Republic of Korea

**Keywords:** Talus, Total extrusion, Reimplantation, Osteonecrosis, Revascularization

## Abstract

**Background:**

Open total extrusion of the talus without concomitant fracture is an extremely rare injury. We present 6-year follow-up data of a patient treated using a temporary spanning external fixator and less invasive single K-wire fixation.

**Case presentation:**

A 55-year-old Asian man who had a totally extruded talus without fracture underwent immediate reimplantation surgery. A spanning external fixator with single antegrade K-wire fixation was applied to maintain the reimplanted talus. During 6 years of follow-up, he could walk without aids and could squat, corresponding to an American Orthopaedic Foot and Ankle Society score of 85. We found that the suspect lesion that was evident at 6 months after surgery had disappeared at 12 months postoperatively on the basis of sequential follow-up magnetic resonance imaging. There was no evidence of osteonecrosis of the dislocated talus at the final follow-up.

**Conclusions:**

In patients with a totally extruded talus, a surgical strategy including immediate reimplantation of the talus and a temporary spanning fixator with single K-wire fixation might be useful to allow early mobilization around the ankle joint and to prevent additional damage of the foot without significant complications.

## Background

Open total extrusion of the talus without concomitant fracture is an extremely rare injury, accounting for 0.06% of all dislocations and 2% of all talar injuries [1–3]. Very few sporadic cases have been reported in the English literature, and there is no definite consensus concerning management. Primary ankle fusion was once recommended as the initial procedure. More recently, reimplantation of a completely extruded talus through the wound has been more recommended, and results have been satisfactory [4–8]. However, fixation constructs that are needed to maintain the reimplanted talus have not been well established. Therefore, we present 6-year follow-up data of a patient treated using a temporary spanning external fixator and less invasive single K-wire fixation. A good clinical outcome featuring revascularization of the extruded talus was attained.

## Case presentation

**Fig. 1:**
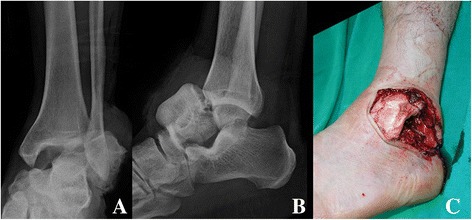
**a** and **b** The talus was completely extruded without significant fracture to the anterolateral direction. **c** The open wound showed a small focal lesion of chondral injury

**Fig. 2:**
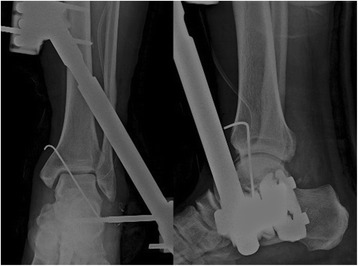
After immediate reimplantation of the extruded talus, its position around the ankle joint was maintained using a single K-wire fixation and spanning external fixator. Postoperative radiographs show the anatomical reduction of the ankle joint, including the subtalar joint and tibiotalar joint

**Fig. 3:**
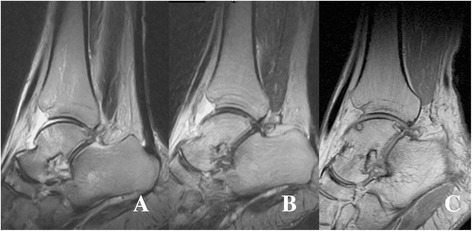
**a** Magnetic resonance imaging scan obtained 6 months after surgery shows high intensity at the anterior aspect of the talar body, which was caused by the vascular insufficiency. **b** After 12 months, the suspected lesion of talus had disappeared and had returned to nearly the normal signal compared with adjacent bones. **c** After 5 years, the reimplanted talus had a small-sized subchondral lesion without osteonecrosis

**Fig. 4:**
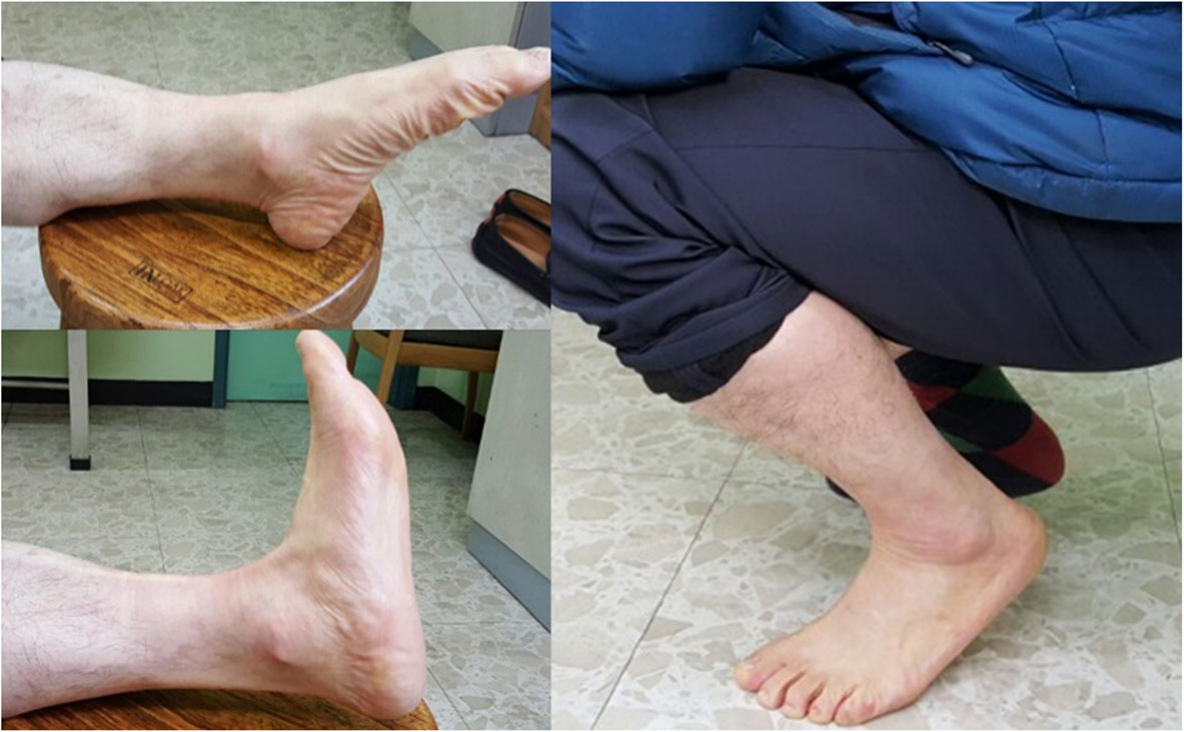
The patient’s condition returned to the preinjury level without motion limitation of the ankle joint, and he was able to squat for 5 minutes with mild pain. His American Orthopaedic Foot and Ankle Society score was 85 (pain 30, function 45, alignment 10)

A 55-year-old Asian man was brought to the emergency department of our institution after a workplace accident in which his ankle had been squashed by heavy steel. The patient did not remember in detail how the incident had happened. At the time of examination, his left talus was totally extruded through a 12-cm anterolateral wound (Fig. 1). Based on the principles of open fracture management [9, 10], urgent debridement of wound and talus reduction, along with early administration of preventive antibiotics as well as a tetanus toxoid booster, was planned as soon as possible. Two hours later, with the patient under general anesthesia, the injured ankle was cleaned and irrigated copiously with sterile normal saline in the operating room. Intraoperative evaluation revealed that the talus was completely extruded from its articulation without any significant fracture and articular cartilage damage, and it was only loosely held by a few remaining strands of the deltoid ligament. The extruded talus was easily reimplanted through the open wound without additional procedures. A single transarticular K-wire was inserted from the proximal medial to distal lateral (medial malleolus to cuboid bone) direction, and a monolateral spanning external fixator was applied to maintain the gap of the joint surface in favor of the articular damage repair (Fig. 2). Finally, the wound was loosely closed. Three days later, second-look surgery for debridement and irrigation was done. After 4 weeks, the external fixator was removed, and weight bearing was forbidden for the first 3 months. The transarticular K-wire was eliminated after another 4 weeks (postoperative day (POD) 2 months). Passive range-of-motion (ROM) exercise and partial weight bearing was started at POD 3 months. At POD 6 months, full weight bearing and active ROM exercise were started. A magnetic resonance imaging (MRI) scan (POD 6 months) showed a substantial signal change between the talus and adjacent bones. The signal change disappeared on subsequent scans done at 12 months and 5 years (Fig. 3). No complications had occurred through 6 years of follow-up, including increased sclerosis of the talar body and joint space narrowing without trivial pain. The patient’s ankle joint ROM was 35 degrees of plantar flexion and 15 degrees of dorsiflexion. The patient could walk without aids and could squat, corresponding to an American Orthopaedic Foot and Ankle Society score of 85, comprising a score of 30 points for pain, 45 for function, and 10 for alignment (Fig. 4).

## Discussion

Total talus extrusion without fracture is an extremely rare and potentially debilitating injury. Few reports have described it [1, 6, 11–13]. Despite the absence of a congruent evidence-based strategy, immediate reimplantation has been considered the first-line treatment. Once the talus is extruded over the skin, surgeons should focus on prevention of infection and fixation constructs, as mandated in the management of open fracture, and the additional risk of osteonecrosis due to talus vascularity [14]. In our patient, the totally extruded talus was managed by immediate reimplantation and minimization of soft tissue damage. The approach produced an excellent outcome and complete revascularization at the 6-year follow-up.

Concerning the fixation constructs for a reimplanted talus, Van Opstal *et al.* [15], Fleming *et al.* [16], and Breccia *et al.* [12] applied only external fixation as the definitive method without talar fixation. Karampinas *et al.* [1] used two Steinmann pins placed from the inferior aspect of the calcaneus through the talus into the inferior aspect of the tibia with a circular external fixator. However, Turhan *et al*. [2] made the fixation construct using two retrograde Kirschner wires, and Apostle *et al.* [4] used a single provisional Kirschner wire. Their outcomes could not be verified, because postoperative radiographs were not included.

No clear strategy for use of fixation constructs has been established. Considering their many advantages, including the elimination of gross movement at the fracture site, improved blood flow, and reduced postoperative edema [10], in one study a spanning external fixator was applied as a temporary option to maintain the joint space around the reimplanted talus to prevent complications, including pin-site infections and joint contracture. In this approach, half-pins were inserted into the distal tibia and calcaneus with careful attention paid to avoid overdistraction. To directly maintain the position of the talus, a single K-wire was placed in an antegrade direction from the proximal medial to the distal lateral aspect of the ankle joint.

Through this less invasive technique, we could prevent additional damage in the plantar area of the foot and open wound, and the patient achieved partial weight bearing before removal of the K-wire. The ankle was actively mobilized as much as possible from 3 weeks postoperatively, and the patient started ambulating with toe-touch walking with crutches after 7 weeks. Despite the early exercise protocol, the patient had no significant pain around the ankle. Prolonged immobilization and offloading is not indicated in cases of asymptomatic osteonecrosis and may lead to a delay of remineralization [17]. So, our surgical strategy might be useful.

**Fig. 5:**
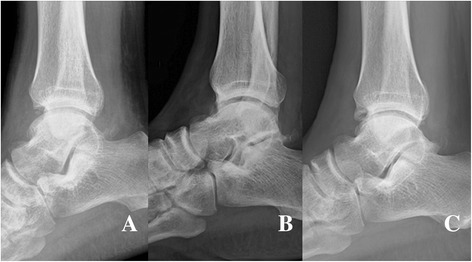
**a** This plain radiograph obtained at 8 weeks postoperatively did not show the Hawkins sign in the reimplanted talus. **b** After 24 months, the anterior osteophyte appeared without narrowing at the ankle joint surface. **c** At the last follow-up at 6 years, the reimplanted talus was well positioned in the ankle joint without avascular necrosis

In talus injury, osteonecrosis and arthritis should always be concerns [18]. In the past, immediate tibiocalcaneal arthrodesis was usually favored over reimplantation [19–21]. However, sporadic reports of favorable outcomes with reimplantation led to the exploration of reimplantation as a first-line treatment [22]. In our patient, for surveillance of osteonecrosis progression, serial MRI was performed at 6 months, 1 year, and 5 years after the injury (Fig. 3). Because there was no Hawkins sign, which is the only early sign that can be seen with conventional radiography and that can reliably predict the development of avascular necrosis (AVN) [23], at 8 weeks after surgery (Fig. 5), we were concerned that the abnormal findings of the first MRI might indicate AVN of the talus. However, the abnormality had disappeared on the 5-year follow-up MRI scan. Based on this finding, and considering that these traumatic injuries tend to affect younger patients [6], the confirmative diagnosis and management of AVN should be delayed as long as possible owing to the possibility of revascularization.

To our knowledge, there has been no report on the revascularization of a completely extruded talus with clear proof by serial MRI. Our findings revealed the possibility of revascularization and change with time of the extruded talus. Our less invasive surgical strategy with early motion exercise, which consists of a temporary spanning external fixator and single antegrade K-wire fixation, might be useful in preventing additional damage of soft tissue around the ankle joint and limb disability due to prolonged immobilization.

## Conclusions

Immediate reimplantation of a totally extruded talus is a well-known salvage procedure. For this surgical strategy, a fixation construct featuring temporary spanning external fixator and less invasive K-wire fixation can avoid soft tissue damage around the ankle.

## References

[1] Karampinas PK, Kavroudakis E, Polyzois V, Vlamis J, Pneumaticos S. Open talar dislocations without associated fractures. Foot Ankle Surg. 2014;20(2):100–4. 10.1016/j.fas.2013.12.005 24796827

[2] Turhan Y, Cift H, Ozkan K, Ozkut A, Eren A. Closed total talar extrusion after ankle sprain. Foot Ankle Spec. 2012;5(1):51–3. 10.1177/1938640011418497 22134439

[3] Mnif H, Zrig M, Koubaa M, Jawahdou R, Hammouda I, Abid A. Reimplantation of a totally extruded talus: a case report. J Foot Ankle Surg. 2010;49(2):172–5. 10.1053/j.jfas.2009.09.003 20015667

[4] Apostle KL, Umran T, Penner MJ. Reimplantation of a totally extruded talus: a case report. J Bone Joint Surg Am. 2010;92(7):1661–5. 10.2106/JBJS.I.00867 20595574

[5] Brewster NT, Maffulli N. Reimplantation of the totally extruded talus. J Orthop Trauma. 1997;11(1):42–5. 10.1097/00005131-199701000-00011 8990033

[6] Gerken N, Yalamanchili R, Yalamanchili S, Penagaluru P, Milman E, Cox G. Talar revascularization after a complete talar extrusion. J Orthop Trauma. 2011;25(11):e107–10. 10.1097/BOT.0b013e318210f236 21577150

[7] Lee J, Hamilton G. Complete talar extrusion: a case report. J Foot Ankle Surg. 2009;48(3):372–5. 10.1053/j.jfas.2009.01.008 19423041

[8] Smith CS, Nork SE, Sangeorzan BJ. The extruded talus: results of reimplantation. J Bone Joint Surg Am. 2006;88(11):2418–24. 10.2106/JBJS.E.00471 17079399

[9] Prodromidis AD, Charalambous CP. The 6-hour rule for surgical debridement of open tibial fractures: a systematic review and meta-analysis of infection and nonunion rates. J Orthop Trauma. 2016;30(7):397–402. 10.1097/BOT.0000000000000573 26978135

[10] Giannoudis PV, Papakostidis C, Roberts C. A review of the management of open fractures of the tibia and femur. J Bone Joint Surg (Br). 2006;88(3):281–9. 10.1302/0301-620X.88B3.16465 16497997

[11] Dumbre Patil SS, Abane SR, Dumbre Patil VS, Nande PN. Open fracture dislocation of the talus with total extrusion: a case report. Foot Ankle Spec. 2014;7(5):427–31. 10.1177/1938640014528040 24686908

[12] Breccia M, Peruzzi M, Cerbarano L, Galli M. Treatment and outcome of open dislocation of the ankle with complete talar extrusion: a case report. Foot (Edinb). 2014;24(2):89–93. 10.1016/j.foot.2014.03.008 24736016

[13] Burston JL, Brankov B, Zellweger R. Reimplantation of a completely extruded talus 8 days following injury: a case report. J Foot Ankle Surg. 2011;50(1):104–7. 10.1053/j.jfas.2010.10.009 21106409

[14] Babu N, Schuberth JM. Partial avascular necrosis after talar neck fracture. Foot Ankle Int. 2010;31(9):777–80. 10.3113/FAI.2010.0777 20880480

[15] Van Opstal N, Vandeputte G. Traumatic talus extrusion: case reports and literature review. Acta Orthop Belg. 2009;75(5):699–704. 19999887

[16] Fleming J, Hurley KK. Total talar extrusion: a case report. J Foot Ankle Surg. 2009;48(6):690–23. 10.1053/j.jfas.2009.06.005 19857829

[17] Rammelt S, Zwipp H. Talar neck and body fractures. Injury. 2009;40(2):120–35. 10.1016/j.injury.2008.01.021 18439608

[18] D’Ambrosi R, Maccario C, Serra N, Liuni F, Usuelli FG. Osteochondral lesions of the talus and autologous matrix-induced chondrogenesis: is age a negative predictor outcome? Arthroscopy. 2017;33(2):428–35. 10.1016/j.arthro.2016.09.030 27956234

[19] Canale ST. Fractures of the neck of the talus. Orthopedics. 1990;13(10):1105–15. 10.3928/0147-7447-19901001-06 2251228

[20] Detenbeck LC, Kelly PJ. Total dislocation of the talus. J Bone Joint Surg Am. 1969;51(2):283–8. 4975068

[21] Hawkins LG. Fractures of the neck of the talus. J Bone Joint Surg Am. 1970;52(5):991–1002. 5479485

[22] Weston JT, Liu X, Wandtke ME, Liu J, Ebraheim NE. A systematic review of total dislocation of the talus. Orthop Surg. 2015;7(2):97–101. 10.1111/os.12167 PMC6583369 26033988

[23] Mohammad T, Clemens D, Klaus MS. Prognostic reliability of the Hawkins sign in fractures of the talus. J Orthop Trauma. 2007;21:538–43. 10.1097/BOT.0b013e318148c665 17805020

